# Preferences for Multipurpose Technology and Non-oral Methods of Antiretroviral Therapy Among Women Living With HIV in Western Kenya: A Survey Study

**DOI:** 10.3389/fgwh.2022.869623

**Published:** 2022-05-19

**Authors:** Caitlin Bernard, Beatrice Jakait, William F. Fadel, A. Rain Mocello, Maricianah A. Onono, Elizabeth A. Bukusi, Kara K. Wools-Kaloustian, Craig R. Cohen, Rena C. Patel

**Affiliations:** ^1^Department of Obstetrics & Gynecology, Division of Family Planning, Indiana University School of Medicine, Indianapolis, IN, United States; ^2^Moi Teaching & Referral Hospital/Moi University & Academic Model Providing Access to Healthcare (AMPATH), Eldoret, Kenya; ^3^Department of Biostatistics, Indiana University School of Medicine, Indianapolis, IN, United States; ^4^Bixby Center for Global Reproductive Health, Department of Obstetrics, Gynecology & Reproductive Health, University of California San Francisco, CA, United States; ^5^Centre for Microbiology Research, Kenya Medical Research Institute, Nairobi, Kenya; ^6^Department of Medicine, Indiana University School of Medicine, Indianapolis, IN, United States; ^7^Division of Allergy and Infectious Diseases, Department of Medicine, University of Washington, Seattle, WA, United States

**Keywords:** contraception, HIV, multipurpose technology, women, antiretroviral therapy

## Abstract

**Introduction:**

Understanding interests in and preferences for multipurpose technology (MPT) for the co-administration of contraception and antiretroviral therapy (ART) and alternative, non-oral ART methods among women living with HIV (WLHIV) is vital to successful implementation of future treatment options, such as long-acting injectable ART.

**Methods:**

Between May 2016 and March 2017 we conducted a cross-sectional telephone survey of 1,132 WLHIV of reproductive potential with prior experience using intermediate- or long-acting contraceptive methods in western Kenya. We present descriptive statistics and multinomial logistic regression to evaluate predictors of interest in specific MPT and non-oral ART methods.

**Results:**

Two-thirds (67%) reported interest in MPT, with the most common reason for interest being ease of using a single medication for both purposes of HIV treatment and pregnancy prevention (26%). Main reasons for lack of interest in MPT were need to stop/not use contraception while continuing ART (21%) and risk of side effects (16%). Important characteristics of MPT were effectiveness for pregnancy prevention (26%) and HIV treatment (24%) and less than daily dosing (19%). Important characteristics of non-oral ART methods were less than daily dosing (47%), saving time accessing ART (16%), and effectiveness of HIV treatment (15%). The leading preferred methods for both MPT and non-oral ART were injectables (50 and 54%) and implants (32 and 31%). Prior use of a contraceptive implant or injectable predicted interest in similar methods for both MPT and non-oral ART methods, while this relationship did not appear to vary between younger vs. older WLHIV.

**Discussion:**

Most WLHIV in western Kenya are interested in MPT for HIV treatment and contraception. Prior exposure to contraceptive implants or injectables appears to predict interest in similar methods of MPT and non-oral ART. Developers of MPT and non-oral ART methods should strongly consider WLHIV's preferences, including their changing reproductive desires.

## Introduction

Women account for greater than half of the estimated 36 million people living with HIV and 2 million new infections annually (1). Strides have been made in achieving universal access to antiretroviral therapy (ART), improving the quality of life and years of reproductive potential for women living with HIV (WLHIV) (2). Similar strides remain to be realized for family planning, especially for WLHIV, who are less likely to use any contraception, more likely to rely on condoms alone, and more likely to experience unintended pregnancy, compared with HIV-negative women (3–5).

Progress has been made in the development of multipurpose technology (MPT) for the delivery of antiretrovirals and contraception for the *prevention* of HIV and pregnancy (6). Similarly, MPT that combines contraception with ART for HIV *treatment* may improve access to and effectiveness of *both* ART and contraceptive use. Such technology could decrease morbidity and mortality related to both HIV and pregnancy, two of the greatest health threats to women of reproductive age (7). User-independent long-acting reversible contraception (LARC), including intrauterine devices (IUDs) and implants, and intermediate-acting injectable methods have higher effectiveness and continuation rates than methods requiring use daily or at every act of intercourse (8, 9). Oral pill ART has similar difficulties (10), thus longer-acting, non-oral ART methods may improve ART adherence and HIV outcomes. As a parallel, injectable pre-exposure HIV prophylaxis shows nine times greater efficacy than oral methods (11).

The HIV treatment field is at an exciting cusp of offering long-acting injectable ART delivery alternatives (12–18), and additional long-acting methods show promising results. Providing an array of options for HIV treatment and pregnancy prevention has the potential to revolutionize personal decision-making and improve long-term HIV and reproductive health outcomes for WLHIV, leading to greater gender equity and women's empowerment.

However, little is known about users' interest in and preferences for MPT and non-oral ART, especially for WLHIV in resource-limited settings, who will be the majority users. Given this gap, our primary objective was to determine the interest in and preferences for future MPT and non-oral ART among WLHIV with prior contraceptive experience in western Kenya. Our secondary objective was to evaluate whether prior use of a specific contraceptive method was associated with preferences for similar MPT and non-oral ART methods; we hypothesized that prior use would positively effect preference for a similar method for MPT and non-oral ART.

## Materials and Methods

### Design

We conducted a cross-sectional telephone survey from May 2016 to March 2017 among WLHIV age 15–45 years at the time of enrollment in care (January 2011–December 2015), whose electronic medical records (EMR) indicated use of an intermediate- or long-acting contraceptive method and who were followed at two large HIV treatment programs in western Kenya affiliated with the East Africa International Epidemiology Databases to Evaluate AIDS (EA-IeDEA). These President's Emergency Plan for AIDS Relief-sponsored HIV treatment programs, the Academic Model Providing Access to Healthcare (AMPATH) and Family AIDS Care & Education Services (FACES), support care for approximately 65,000 and 43,000 HIV-positive individuals in western Kenya, respectively (19, 20).

### Study Population and Sampling

**Figure 1:**
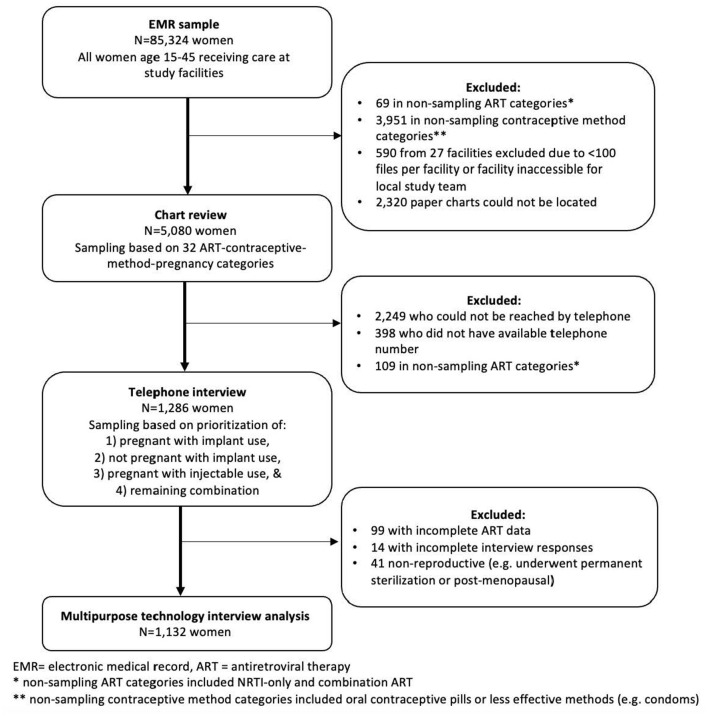
Participant sampling flow diagram.

This telephone survey was part of a larger, 3-phase sampling study to validate exposures of contraceptive use and ART and outcome of incident pregnancy at AMPATH and FACES (Figure 1). Details of the sampling methods have been published elsewhere (21). Briefly, the initial study cohort of 85,324 participants consisted of all women age 15–45 receiving care at study facilities. Routine clinical and demographic data were collected at the program-supported health facilities at enrollment and follow-up visits utilizing standardized, paper instruments, which trained data clerks transcribed into EMR systems supported by an OpenMRS platform. From the first-phase sample, we sampled a random subset of observations for the second phase for the manual chart review based on a combination of 32 exposure and outcome categories. Our second-phase sample consisted of ~7% and 12% of the women contributing data to the first phase from AMPATH and FACES, respectively. We successfully conducted a chart review for 2,455 of 3,643 (67.4%) and 2,625 of 3,757 (69.9%) of the index observations, i.e., the combination exposure and outcome category that was chosen for any given woman, sampled for the second phase for AMPATH and FACES, respectively, for a total of 5,080 charts reviewed. From the chart reviews that were successfully completed for the second phase, we conducted telephone interviews with a non-random subset of the women. These women were selected for telephone interview based on the index observation in the first sample. The index observations where a woman was noted to be pregnant while using an implant, regardless of ART, were given the first priority in attempting phone calls. The second priority group was the index observations where a woman was noted not to be pregnant while using an implant, regardless of ART. The third and fourth priority groups were the index observations where the woman was noted to be using injectable contraception and pregnant or not pregnant, respectively. The study team generated periodic lists of priority second-phase sample participants to call for the telephone interviews as the chart reviews progressed based on these contraceptive-ART-pregnancy categories. In total we were able to reach and interview 1,286 women. We excluded 99 due to incomplete ART regimen information, 14 due to incomplete responses, and 41 due to being non-reproductive (i.e., postmenopausal or underwent permanent sterilization), leaving 1,132 women for inclusion in this analysis.

### Telephone Interview

Both male and female research assistants with a higher education diploma or degree and additional training specific to this interview guide performed the interviews. They were bi- or trilingual native speakers and conducted the interviews in English, Kiswahili, or Dholuo, based on the participant's preference. The interview questions and responses were translated from English to Kiswahili and Dholuo and back-translated to confirm accuracy. Interviewers identified themselves as research staff associated with the clinical care program in which the client was enrolled. Interview participants underwent verbal informed consent. The interview consisted of a series of questions to confirm the contraceptive method, ART, and pregnancy history during the study observation period. Baseline reproductive and socio-demographic data collected during the EMR and chart reviewed were not confirmed. Next, the interviewer asked a series of questions about MPT prefaced by the statement: “Someday HIV medication and family planning methods may be combined into one medication to both treat HIV and prevent pregnancy.” Participants were asked whether they would be interested in such a medication and reasons why or why not. Similarly, the interviewer asked a series of questions about non-oral methods of ART prefaced by the statement: “Someday HIV medication may come in a different form, such as an injection you take every few weeks, instead of a pill you take every day.” Preferences for MPT and non-oral ART methods were elicited with respondents given options including injectable, implant, transdermal patch, intrauterine device (IUD, also known as “coil”), vaginal ring, pill, and other, and asked to provide their first, second and third choices. Least preferred MPT methods were also elicited along with reasons for lack of interest in the method(s).

Participants were then asked about the most important aspect of a future MPT or non-oral ART method, and asked to rank their first, second and third choices. If they did not provide an unprompted answer, they were prompted with example characteristics from the following list: dosing less frequently than daily, effectiveness of HIV treatment, effectiveness of pregnancy prevention (for MPT), privacy/concealability, safety, and lack of side effects. Lastly, participants were queried regarding their preferences for integration of HIV and contraception care, and disclosure of HIV status to contraception providers and contraceptive use to HIV providers when care is not integrated.

### Analytic Framework

After data collection, prior to analysis, we interpreted and characterized reasons for interest/lack of interest in MPT and important characteristics of MPT and non-oral ART based on the analytic framework developed by Wyatt et al. (22) This framework is based on a thorough review of patients' values and preferences for important attributes of contraceptive methods and categorizes potential attributes influencing contraceptive choice in order to guide contraceptive decision aid development. We hypothesize that these values and preferences will be important to consider in MPT product development to ensure successful implementation and use. Major categories within this framework are: (1) method effect, (2) mechanistic, (3) practical, and (4) social/normative characteristics. Method effects include efficacy for HIV treatment and pregnancy prevention, non-contraceptive health benefits, and side effects. Mechanistic characteristics include how the method is used, including required user action. Practical characteristics include availability and cost. Social/normative characteristics include internal and external influences on use.

### Statistical Analysis

We described socio-demographic and reproductive characteristics of study participants using descriptive statistics. We use frequency tables to describe the reasons for interest/lack of interest in MPT, importance of characteristics of MPT and non-oral ART methods, and method preferences. To determine whether previous implant and/or injectable contraceptive use (binary variable) was associated with implant and/or injectable method preference for the first choice of MPT and non-oral ART (binary variable), we used multinomial logistic regression to calculate adjusted odds ratios of stated method choice. Each model was adjusted for HIV care program, age, education level, marital status, number of living children, most recent ART regimen, and World Health Organization (WHO) clinical stage of HIV disease. We chose these covariates based on an *a priori* assessment of their potential as confounders (3, 23, 24) and their availability within our dataset. To make the coefficients in the models estimable, we combined preferences with low numbers into larger categories. To account for bias created by the missing data for education level, marital status, and number of living children, we conducted multiple imputations by iterative chain estimates. To better understand any differences between younger (ages 15–24) and older (ages > 25) WLHIV, we conducted subgroup analyses with our regression models comparing younger vs. older study participants. All analyses were performed using R version 3.3.3. (25).

## Results

### Baseline Characteristics

The median age at time of interview was 35 years (Table 1). The majority were married/cohabitating (56%), had at least one living child (69%), and reported previous or current contraceptive use (94%), including implants (67%), injectables (48%), pills (7%), and IUDs (2%). Most women were taking either efavirenz or nevirapine-containing ART regimens (85%) and had a recent WHO Stage 1 or 2 disease status (72%).

**Table 1:** Socio-demographic and reproductive characteristics of women of reproductive potential living with HIV with prior experience using effective contraceptive methods who participated in a telephone interview regarding interest in and preferences for multipurpose technology (MPT) and non-oral antiretroviral therapy (ART) methods (*N* = 1,132).

**Characteristic**	***N*** **(%)**
**HIV care program**	
FACES	694 (61.3)
AMPATH	438 (38.7)
**Age, in years**	34.6 (19–51)
**Education level**	
Less than primary	313 (27.7)
Completed primary	245 (21.6)
Completed secondary	136 (12.0)
Missing	438 (38.7)
**Marital status**	
Married or cohabitating	629 (55.6)
Single, separated, or widowed	279 (24.6)
Missing	224 (19.8)
**Living children**	
1	106 (9.4)
2	272 (24.0)
3	211 (18.6)
4+	189 (16.7)
Missing	354 (31.3)
**ART regimen**	
Efavirenz-based	496 (43.8)
Nevirapine-based	467 (41.3)
Protease-inhibitor-based	117 (10.3)
No ART	32 (2.8)
Missing	5 (0.4)
WHO clinical stage^*^	
1	512 (45.2)
2	305 (26.9)
3	258 (22.8)
4	55 (4.9)
Missing	2 (0.2)
Previous contraceptive method(s) used^**^	
Implant	756 (66.8)
Injectable	544 (48.1)
Male condoms	138 (12.2)
Oral contraceptive pills	79 (7.0)
Intrauterine device	27 (2.4)
Lactational amenorrhea	10 (0.9)

*Values expressed as median (interquartile range) for continuous variables and number of subjects (percentage) for categorical variables. FACES, Family AIDS Care and Education Services; AMPATH, Academic Model Providing Access to Healthcare; ART, antiretroviral therapy*.

* *World Health Organization Clinical Stage of HIV at most recent clinic visit*.

** *Total exceeds 100% due to previous use of multiple methods*.

### Multipurpose Technology (MPT)

#### Interest in MPT and Reasons for Interest or Disinterest

**Figure 2:**
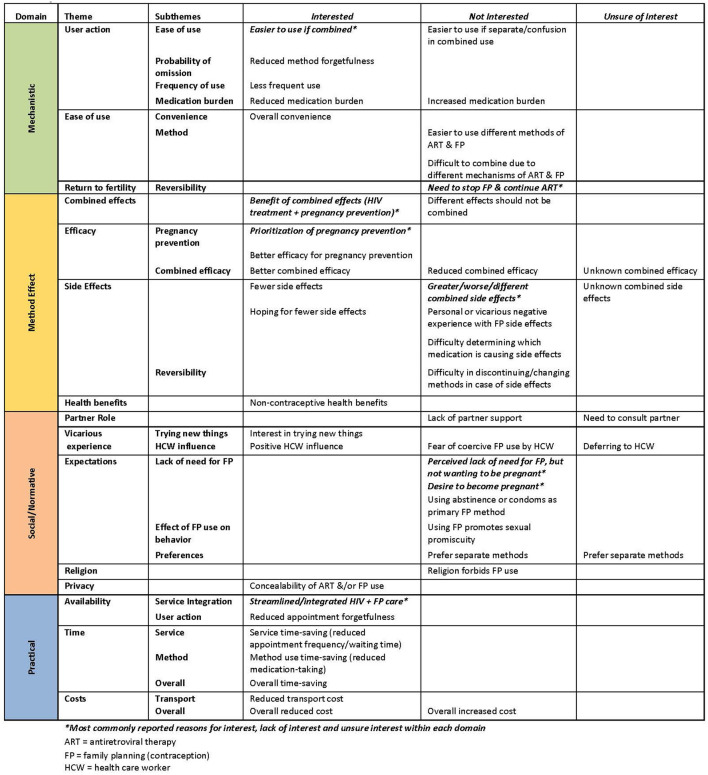
Reported reasons for interest, lack of interest, and unsure interest in the use of multipurpose technology (MPT) for contraception and antiretroviral (ART) therapy among women of reproductive potential living with HIV (*N* = 1132). Qualitative interpretation classified based on the analytic framework of Wyatt, et al. for the categorization of attributes influencing contraceptive choice.

Two-thirds of participants (760, 67%) reported interest in MPT for combined ART and contraception. All responses to reasons for interest, disinterest, or “unsure” interest were classified according to Wyatt et al.'s analytic framework (Figure 2). The most commonly cited positive method effect characteristics were for pregnancy prevention (122, 16%) and to both treat HIV and prevent pregnancy (combined effects, 114, 15%). The most commonly cited positive mechanistic characteristic was ease of use of one medication for both purposes (194, 26%). The most commonly cited positive practical reason was to receive HIV and contraceptive care at the same site (service integration, 92, 12%).

Of the 350 women not interested in MPT, the most commonly cited negative effect characteristic was risk of increased, worse, or different side effects related to a combined medication (55, 16%). The most commonly cited negative mechanistic characteristic was the potential need to stop or not use contraception while continuing ART (reversibility, 75, 21%). The most commonly cited negative social/normative characteristics were perceived lack of need for contraception despite not desiring pregnancy (43, 12%) and desiring pregnancy (29, 8%)

#### Preferences for MPT Methods

The most important reported characteristics of a potential MPT method were effectiveness of pregnancy prevention (289, 26%), effectiveness of HIV treatment (274, 24%) and less than daily dosing (218, 19%) (Table 2). Privacy/concealability, method safety, lack of side effects, and time-saving were lower priority characteristics. The most commonly cited first and second choice MPT methods were injectables (560, 50%; 159, 14%) and implants (364, 32%; 280, 25%) (Table 3). Least preferred MPT methods were the intrauterine device (354, 31%), pills (31, 28%), and vaginal ring (228, 20%). Common reasons cited for lack of interest in all methods were fear of side effects/complications and previous negative personal or vicarious experiences, including bleeding, pain, and contraceptive method failure.

**Table 2:** Importance of characteristics of methods of multipurpose technology (MPT) for contraception and antiretroviral therapy (ART) and non-oral ART reported among women of reproductive potential living with HIV (*N* = 1,132).

**Multipurpose**	**First choice**	**Second choice**	**Third choice**
**technology**	***n*** **(%)**	***n*** **(%)**	***n*** **(%)**
**Method effect**	289 (25.5)	239 (21.1)	108 (9.5)
Effectiveness for pregnancy prevention			
Effectiveness for HIV treatment	274 (24.2)	249 (22.0)	108 (9.5)
Safety	61 (5.4)	71 (6.3)	88 (7.8)
Lack of side effects	52 (4.6)	59 (5.2)	45 (4.0)
**Mechanistic**	218 (19.3)	129 (11.4)	153 (13.5)
Dosing less frequently than daily			
**Social/normative**	101 (8.9)	116 (10.2)	129 (11.4)
Privacy/concealability			
**Practical**	41 (3.6)	20 (1.8)	29 (2.6)
Save time accessing medication			
Other^*^	26 (2.3)	7 (0.6)	9 (0.8)
Missing	70 (6.2)	242 (21.4)	463 (40.9)
**Non-oral**	**First choice**	**Second choice**	**Third choice**
**ART**	***n*** **(%)**	***n*** **(%)**	***n*** **(%)**
**Mechanistic**	532 (47.0)	198 (17.5)	88 (7.8)
Dosing less frequently than daily			
**Practical**	177 (15.6)	46 (4.1)	24 (2.1)
Save time accessing medication			
**Method effect**	173 (15.3)	178 (15.7)	158 (14.0)
Effectiveness for HIV treatment			
Safety	57 (5.0)	115 (10.2)	123 (10.9)
Lack of side effects	71 (6.3)	75 (6.6)	91 (8.0)
**Social/Normative**	83 (7.3)	186 (16.4)	151 (13.3)
Privacy/concealability			
Other^*^	39 (3.4)	13 (1.1)	7 (0.6)
Missing	0	321 (28.4)	490 (43.3)

* *Other characteristics include method effect (e.g., effectiveness of combined medication, other health benefits), mechanistic (e.g., ease of use, reduced frequency of use, reduced frequency of forgetting medication, smaller pill size, reduced pill burden, not having to carry medications, not being a foreign body or food-dependent), practical (e.g., reducing appointment frequency, reducing costs) & social-normative (e.g., previous experience, reduced stress)*.

**Table 3:** Most and least preferred methods of multipurpose technology (MPT) for contraception and antiretroviral therapy (ART) and non-oral ART reported among women of reproductive potential living with HIV (*N* = 1,132).

**Multipurpose**	**First**	**Second**	**Third**	**Least**
**technology**	**choice**	**choice**	**choice**	preferred^*^
	***n*** **(%)**	***n*** **(%)**	***n*** **(%)**	***n*** **(%)**
Injectable	560 (49.5)	159 (14.0)	70 (6.2)	81 (7.2)
Implant	364 (32.2)	280 (24.7)	113 (10.0)	79 (7.0)
Pill	81 (7.2)	117 (10.3)	161 (14.2)	318 (28.1)
Transdermal patch	33 (2.9)	99 (8.7)	135 (11.9)	106 (9.4)
Intrauterine device	25 (2.2)	62 (5.5)	121 (10.7)	360 (31.8)
Vaginal ring	1 (0.1)	6 (0.5)	11 (1.0)	222 (19.6)
Other^**^/unsure	0	3 (0.3)	13 (1.1)	8 (0.7)
Missing	68 (6.0)	406 (35.9)	508 (44.9)	40 (3.5)
**Non-oral**	**First choice**	**Second choice**	**Third choice**	
**ART**	***n*** **(%)**	***n*** **(%)**	***n*** **(%)**	
Injectable	607 (53.6)	181 (16.0)	61 (5.4)	
Implant	350 (30.9)	231 (20.4)	149 (13.2)	
Pill	105 (9.3)	132 (11.7)	152 (13.4)	
Transdermal patch	42 (3.7)	125 (11.0)	136 (12.0)	
Intrauterine device	14 (1.2)	49 (4.3)	94 (8.3)	
Vaginal ring	1 (0.1)	7 (0.6)	14 (1.2)	
Other^**^/unsure	10 (0.9)	10 (0.9)	15 (1.4)	
Missing	3 (0.3)	397 (35.1)	511 (45.1)	

* *Total exceeds 100% due to multiple potential responses per participant*.

** *Other includes chewable tablet, candy form, food additive, syrup, and topical methods*.

#### Predictors of MPT Preferences

Women who previously used a contraceptive implant were more likely to be interested in an implant for MPT compared to both injectables (aOR 2.65, 95% CI 1.74–4.04) and pills (aOR 3.16, 95% CI 1.48–6.71) (Table 4). Similarly, women who previously used injectable contraception were more likely to be interested in an injectable for MPT compared to an implant (aOR 1.69, 95% CI 1.20–2.44). There was no significant difference in preference for implant compared to injectables or pills by younger vs. older age (aOR 1.67, 95% CI 0.69–4.03 and aOR 0.62, 95% CI 0.15–2.51, respectively). Women enrolled in AMPATH and those who completed secondary education were more likely to prefer implants over injectables (aOR 1.99, 95% CI 1.37–2.88 and aOR 2.89, 95% CI 1.58–5.27, respectively). There was no statistically significant relationship between other measured covariates and preferred method for MPT.

**Table 4:** Results of multinomial logistic regression to determine factors associated with preferred method of multipurpose technology (MPT) for contraception and antiretroviral therapy (ART) and method of non-oral ART (*N* = 1,132).

**Characteristic**	**Preferred MPT method**		**Preferred non-oral ART method**	
	**Implant vs. injectable**	**Implant vs. pills**	**Implant vs. injectable**	**Implant vs. pills**
**Previous implant use**				
No	*Ref*	*Ref*	*Ref*	*Ref*
Yes	2.65 (1.74–4.04)^*^	3.16 (1.48–6.71)^*^	**1.88 (1.35–2.63)**	2.27 (1.38–3.76)^*^
**Previous injectable use**				
No	*Ref*	*Ref*	*Ref*	*Ref*
Yes	0.59 (0.41–0.83)^*^	1.03 (0.51–2.09)	0.56 (0.42–0.76)^*^	0.85 (0.53–1.36)
**Age**				
>25 y	*Ref*	*Ref*	*Ref*	*Ref*
<25 y	1.67 (0.69–4.03)	0.62 (0.15–2.51)	1.69 (0.81–3.53)	0.81 (0.32–2.09)
**Program**				
FACES	*Ref*	*Ref*	*Ref*	*Ref*
AMPATH	1.99 (1.37–2.88)^*^	1.55 (0.72–3.33)	2.08 (1.51–2.86)^*^	3.04 (1.74–5.30)^*^
**Education**				
Less than primary	*Ref*	*Ref*	*Ref*	*Ref*
Completed primary	1.24 (0.78–1.97)	1.55 (0.52–4.59)	1.20 (0.83–1.74)	1.54 (0.81–2.91)
Completed secondary	2.89 (1.58–5.27)^*^	1.72 (0.55–5.36)	2.25 (1.39–3.63)^*^	1.51 (0.67–3.44)
	**Preferred MPT method**		**Preferred non-oral ART method**	
	**Injectable vs. implant**	**Injectable vs. pills**	**Injectable vs. implant**	**Injectable vs. pills**
**Previous implant use**				
No	*Ref*	*Ref*	*Ref*	*Ref*
Yes	0.38 (0.25–0.57)	1.19 (0.58–2.44)	0.53 (0.38–0.74)	1.21 (0.77–1.90)
**Previous injectable use**				
No	*Ref*	*Ref*	*Ref*	*Ref*
Yes	1.69 (1.20–2.44)^*^	1.76 (0.88–3.50)	1.79 (1.32–2.38)^*^	1.52 (0.98–2.35)
**Age**				
>25 y	*Ref*	*Ref*	*Ref*	*Ref*
<25 y	0.60 (0.25–1.45)	0.37 (0.09–1.48)	0.60 (0.28–1.23)	0.48 (0.20–1.16)
**Program**				
FACES	*Ref*	*Ref*	*Ref*	*Ref*
AMPATH	0.50 (0.35–0.73)^*^	0.78 (0.37–1.66)	0.48 (0.35–0.66)^*^	1.46 (0.86–2.50)
**Education**				
Less than primary	*Ref*	*Ref*	*Ref*	*Ref*
Completed primary	0.81 (0.51–1.28)	1.26 (0.44–3.58)	0.83 (0.57–1.20)	1.28 (0.70–2.31)
Completed secondary	0.35 (0.19–0.63)^*^	0.60 (0.19–1.88)	0.44 (0.28–0.72)^*^	0.67 (0.29–1.55)

*Reported as Adjusted Odds Ratio (95% Confidence Interval)*.

* *Statistically significant at p < 0.005*.

*NB: There were insufficient numbers of women who previously used or preferred other methods to draw statistical conclusions. Bold values indicates Statistically significant at p < 0.005*.

### Non-oral Methods of Antiretroviral Therapy (ART)

#### Interest in Non-oral Methods of ART and Reasons for Interest

The most common first and second choices for non-oral ART methods were injectables (607, 54%; 181, 16%) and implants (350, 31%; 231, 20%) (Table 3). The most important characteristics of non-oral ART methods were less than daily dosing (532, 47%), saving time accessing it (177, 16%), and effectiveness of HIV treatment (173, 15%). Privacy/concealability, method safety, and lack of side effects were lower priorities.

#### Predictors of Non-oral ART Method Preference

Women who previously used an implant for contraception were also more likely to be interested in an implant for non-oral ART compared to both injectables (aOR 1.88, 95% CI 1.35–2.63) and pills (aOR 2.27, 95% CI 1.38–3.76) (Table 4). Similarly, women who previously used injectable contraception were more likely to be interested in an injectable for non-oral ART compared to an implant (aOR 1.79, 95% CI 1.32–2.38). There was no significant difference in preference for implant compared to injectables or pills by younger vs. older age (aOR 1.69, 95% CI 0.81–3.53 and aOR 0.81, 95% CI 0.32–2.09, respectively). Women enrolled in AMPATH and those who completed secondary education were more likely to prefer implants over injectables (aOR 2.08, 95% CI 1.51–2.86 and aOR 2.25, 95% CI 1.39–3.63, respectively). There was no statistically significant relationship between other measured covariates and preferred method for non-oral ART.

#### Dosing Frequency and Preference for Site of HIV and Contraception Care

Most participants preferred annual or semi-annual dosing for both MPT and ART (610, 54%; and 680, 60%, respectively). Most participants preferred to receive integrated contraceptive and HIV services at the same site (865, 76%). The majority of participants reported disclosing contraceptive use to their HIV care provider (1,111, 98%), while fewer reported disclosing their HIV status to their contraception provider (925, 82%). Of the 171 women who reported not disclosing HIV status to a contraception provider, the most commonly cited reasons were not using contraception (31, 18%), feeling that it was unnecessary to disclose HIV status when obtaining contraception (22, 13%), and discomfort in disclosing HIV status (22, 13%).

## Discussion

### Main Findings

Our study is the first to elicit values and preferences regarding potential multipurpose technology for ART and contraception and non-oral ART among WLHIV in a resource-limited setting. We find that the majority of WLHIV who have some experience with injectable or long-acting reversible contraception in western Kenya are interested in the use of such MPT, which is promising for the advancement of the MPT field for those already living with HIV, and not just for women at risk of acquiring HIV (26). Additionally, the high interest in implants and injectables for both MPT and non-oral ART are important findings to guide product development and scale-up, especially now that injectable ART has been approved in resource-rich settings and is on the cusp of approval in resource-limited settings (27).

### Interpretation

The development of a target product profile (TPP) that identifies desired method parameters, characteristics of the user population, and the environment influencing acceptability are important to guide product development and implementation (28). The Wyatt et al. framework we use offers an approach that prioritizes user values and preferences for contraceptive methods that are vital to incorporate into the TPP for MPT and non-oral ART in order to achieve sustainability of these technologies. Our findings highlight similarities in the TPP for both non-oral ART and MPT, including the effectiveness of pregnancy prevention and HIV treatment, less than daily dosing, and methods that save time for users. Our data also indicates the importance of emphasizing the co-benefits and the ease of use of one product for both pregnancy prevention and HIV treatment, including service integration to improve simultaneous access to HIV and family planning services. This reinforces previous work that has shown high client satisfaction and improved contraceptive access with integrated services (29). At the same time, attention needs to be paid to user concerns about contraceptive reversibility while maintaining ART effectiveness. Implementation plans will need to emphasize respect for women who choose not to use contraception or MPT. The development of MPT and non-oral ART can be conceptualized within the contraceptive method mix framework, with multiple options allowing women to choose the best fit based on their values and preferences, thus improving uptake, continuation, and effectiveness (30). Additionally, preferences vary over time, as reproductive intentions change, and both across and within regions (31), stressing the importance of understanding the preferences and values of the target population early in the TPP process.

We found that prior use of an implant or injectable contraception was associated with interest in a similar method for both MPT and non-oral ART, consistent with recent findings that women's prior contraceptive method use was predictive of preference for a preventive MPT method (32). Notably, most women in our study preferred implants or injectables regardless of previous method use, indicating the opportunity for successful implementation of long-acting ART. We did not find any significant differences in method preference by age, indicating that there may be wide appeal for long-acting MPT and non-oral ART. Similar to findings about preferred preventative MPT (33), the vaginal ring was one of the least preferred methods. This may be reflective of a lack of knowledge or experience with this method, including potential transfer of concerns about IUDs to vaginal rings. This is an important finding given the significant investment and research to-date into vaginal rings for MPT (34). While we advocate for a wide method mix for MPT, questions remain about the acceptability of vaginal rings and how implementation strategies may improve acceptability, and greater emphasis should focus on the development of other methods, including injectables and implants.

### Strengths and Limitations

While this is the first survey of its kind, our study has several limitations. Our study sample was largely WLHIV with experience with intermediate- or long-acting contraceptives due to the nature of the parent study sampling design and comes from HIV treatment programs that have made considerable efforts in integrating contraception into HIV services, limiting the generalizability of our findings other settings. Our data set was missing a relatively large percentage of key sociodemographic confounders, e.g., pregnancy intentions, that may impact the outcomes assessed. The study utilized multiple interviewers and we did not assess for potential inter-interviewer variability that may have contributed to participant responses. Finally, the analytic framework we utilized may not capture all preferences and values of all potential populations, and we hope this provides impetus for the development of new analytic frameworks.

## Conclusion

This study furthers our understanding about the preferred characteristics of methods for MPT and non-oral ART to inform the development and implementation of this important technology for WLHIV. The majority of WLHIV in western Kenya appear interested in MPT and non-oral ART injectable and implantable methods, and want them to be easily reversible. This interest, especially for women experienced with similar methods, signals opportunities for improved adherence to ART and use of effective contraception through longer-acting methods compared to daily pills. Importantly, combining HIV treatment and contraception in one delivery mechanism mirrors the benefits seen for integration of HIV and contraceptive service access. Prioritizing the values and preferences of WLHIV must be central to the product development and implementation for MPT and non-oral ART to maximize uptake and integration into care.

## Data Availability Statement

The raw data supporting the conclusions of this article will be made available by the authors, without undue reservation.

## Ethics Statement

The studies involving human participants were reviewed and approved by Moi Teaching and Referral Hospital/Moi University Institutional Research and Ethics Committee (IREC). Written informed consent for participation was not required for this study in accordance with the national legislation and the institutional requirements.

## Author Contributions

CB assisted with research conception and study design and led implementation at AMPATH. BJ provided assistance with study development and implementation at AMPATH. WF provided assistance with data analysis. AM provided assistance with study development and implementation and data management. MO provided assistance with study development and implementation at FACES. EB provided assisted with study development and implementation at FACES. KW-K assisted with early study conceptualization and reviewed and edited the manuscript. CC helped conceive of and implement this research, and reviewed and edited this manuscript. RP conceived of the research question and study design, led implementation of the study, including data collection, and data analysis. All listed authors made substantial contributions to the conception or design of the work or the acquisition, analysis, or interpretation of data for the work drafted or critically revised the work, submitted final approval of the version to be published, and agree to be accountable for all aspects of the work in ensuring that questions related to the accuracy or integrity of any part of the work are appropriately investigated and resolved.

## Funding

CB received support for this research from the Clinical and Translational Science Award (CTSA) program of the National Center for Advancing Translational Sciences (NCATS) of the National Institutes of Health (NIH) under Award# UL1 TR000448 and TL1 TR000449 and NIH Reproductive Epidemiology Training Grant# T32HD055172. RP received support for this research from the National Institute of Health National Institute of Allergy and Infectious Diseases (K23AI120855). Research reported in this publication was supported by the National Institute of Allergy and Infectious Diseases (NIAID), Eunice Kennedy Shriver National Institute of Child Health & Human Development (NICHD), National Institute on Drug Abuse (NIDA), National Cancer Institute (NCI) and the National Institute of Mental Health (NIMH), in accordance with the regulatory requirements of the National Institutes of Health for the EA-IeDEA Consortium (U01AI069911). This grant included external peer review for scientific quality; the funder was not involved in the conduct of research or manuscript writing.

## Conflict of Interest

The authors declare that the research was conducted in the absence of any commercial or financial relationships that could be construed as a potential conflict of interest.

## Publisher's Note

All claims expressed in this article are solely those of the authors and do not necessarily represent those of their affiliated organizations, or those of the publisher, the editors and the reviewers. Any product that may be evaluated in this article, or claim that may be made by its manufacturer, is not guaranteed or endorsed by the publisher.
