# Supplementary material for: Preferences for Multipurpose Technology and Non-oral Methods of Antiretroviral Therapy Among Women Living With HIV in Western Kenya: A Survey Study
Source: Front Glob Womens Health. 2022 May 19;3:869623. doi: 10.3389/fgwh.2022.869623 (PMC9160913; doi:10.3389/fgwh.2022.869623)
Supplement: Supplementary file 1 [file Data_Sheet_1.PDF]

Client Interview

Record ID - do not change ______________________

Today's date (dd/mm/yyyy) ______________________

Name/initials of person conducting interview/completing this form ______________________

Someday ART may come in a different form, such as an injection you take every few weeks, instead of a pill you take every day. If ART could be available in any form of a medication, how would you prefer to receive such a medication?

	Choice #1	Choice #2	Choice #3
By pill	○	○	○
By injection	○	○	○
By a patch that sticks to your skin	○	○	○
By an implant inserted under your skin	○	○	○
Other (specify below)	○	○	○

Other (specify): ______________________

How often would you prefer to receive ART in an ideal world?

- ○ Every day
- ○ Every week
- ○ Every month or every few months
- ○ Every year or every few years

What would be the most important aspect of this medication to you?

	Choice #1	Choice #2	Choice #3
How effectively it treats HIV	○	○	○
How safe it is	○	○	○
That it does not have any bothersome side effects	○	○	○
That nobody would know you are taking it	○	○	○
That you don't have to remember to take it every day	○	○	○
Other (specify below)	○	○	○

Other (specify): ______________________

Someday ART and family planning methods may be combined into one medication to both treat HIV and prevent pregnancy.

Would you be interested in having your ART and family planning combined together into one medication?

- ◯ Yes
- ◯ No
- ◯ Don't know

Why or why not?

____

If they are combined into one medication, how would you prefer to receive such a medication?

	1	2	3
By pill	◯	◯	◯
By injection	◯	◯	◯
By a patch that is placed on your skin	◯	◯	◯
By a plastic ring that is placed in your vagina	◯	◯	◯
By a plastic or metal coil that is placed inside your uterus	◯	◯	◯
By an implant inserted under your skin	◯	◯	◯
Other (specify below)	◯	◯	◯

Please specify:

____

How would you NOT want to receive such a medication?

- ☐ A pill
- ☐ An injection
- ☐ A patch that is placed on your skin
- ☐ A plastic ring that is placed in your vagina
- ☐ An implant inserted under your skin
- ☐ Other

Please specify:

____

Why not?

____

How often would you prefer to take such a combined medication in an ideal world?

- ◯ Every day
- ◯ Every week
- ◯ Every month or every few months
- ◯ Every year or every few years

What would be the most important thing about this medication to you?

	Choice #1	Choice #2	Choice #3
How effectively it prevents pregnancy	◯	◯	◯
How effectively it treats HIV	◯	◯	◯
How safe it is	◯	◯	◯
That it does not have bothersome side effects	◯	◯	◯
That nobody would know you are taking it	◯	◯	◯
That you don't have to remember to take it every day	◯	◯	◯
Other reason (specify below)	◯	◯	◯

Specify other: ______________________________

Where do you prefer to get FP care?

◯ At the same facility where I get my HIV care
◯ At a public FP facility (like a MOH ANC facility)
◯ At a private FP facility
◯ Other (specify below)

Specify other: ______________________________

Have you told your HIV provider what you are using for FP?

◯ Yes
◯ No

Why not? ______________________________

Have you told your FP provider your HIV status?

◯ Yes
◯ No

Why not? ______________________________

Please enter any additional information here. ______________________________
